# Faces of Pain in Dementia: Learnings From a Real-World Study Using a Technology-Enabled Pain Assessment Tool

**DOI:** 10.3389/fpain.2022.827551

**Published:** 2022-02-22

**Authors:** Mustafa Atee, Kreshnik Hoti, Paola Chivers, Jeffery D. Hughes

**Affiliations:** ^1^The Dementia Centre, HammondCare, Osborne Park, WA, Australia; ^2^Curtin Medical School, Faculty of Health Sciences, Curtin University, Bentley, WA, Australia; ^3^Division of Pharmacy, Faculty of Medicine, University of Pristina, Prishtina, Kosovo; ^4^Institute for Health Research, The University of Notre Dame Australia, Fremantle, WA, Australia; ^5^School of Medical and Health Sciences, Edith Cowan University, Joondalup, WA, Australia

**Keywords:** pain, dementia, facial expressions, action units, artificial intelligence, PainChek®, aged care, real-world

## Abstract

Pain is common in people living with dementia (PLWD), including those with limited verbal skills. Facial expressions are key behavioral indicators of the pain experience in this group. However, there is a lack of real-world studies to report the prevalence and associations of pain-relevant facial micro-expressions in PLWD. In this observational retrospective study, pain-related facial features were studied in a sample of 3,144 PLWD [mean age 83.3 years (SD = 9.0); 59.0% female] using the *Face* domain of PainChek®, a point-of-care medical device application. Pain assessments were completed by 389 users from two national dementia-specific care programs and 34 Australian aged care homes. Our analysis focused on the frequency, distribution, and associations of facial action units [AU(s)] with respect to various pain intensity groups. A total of 22,194 pain assessments were completed. Of the AUs present, AU7 (eyelid tightening) was the most frequent facial expression (48.6%) detected, followed by AU43 (closing eyes; 42.9%) and AU6 (cheek raising; 42.1%) during severe pain. AU20 (horizontal mouth stretch) was the most predictive facial action of higher pain scores. Eye-related AUs (AU6, AU7, AU43) and brow-related AUs (AU4) were more common than mouth-related AUs (e.g., AU20, AU25) during higher pain intensities. No significant effect was found for age or gender. These findings offer further understanding of facial expressions during clinical pain in PLWD and confirm the usefulness of artificial intelligence (AI)-enabled real-time analysis of the face as part of the assessment of pain in aged care clinical practice.

## Introduction

Pain is a common experience in older people living with dementia (PLWD) because of aged-related physical and functional decline and their associated multimorbidity (1). If it remains un(der)treated, pain may lead to multiple adverse health outcomes including behavioral disturbances (e.g., agitation), physical decline (increased frailty), cognitive decline, inappropriate pharmacotherapy, hospitalizations, institutionalization, disabilities, and premature death (2–4). As dementia advances, the ability to verbally communicate the pain experience is diminished. This verbal limitation makes this population more reliant on non-verbal display of actions, such as communicative pain behaviors (e.g., facial expressions and para-lingual vocalizations), to express their pain experience. Clinical and experimental evidence suggest that facial expressions are key behavioral cues to manifest pain in PLWD (5). Facial expressions are known to be dynamic, spontaneous, versatile, and encodable behaviors (6). These criteria allow facial expressions to be contextually meaningful and unique, and hence well placed as a prime target for pain assessments in clinical settings.

The clinical guidelines on persistent pain developed by the American Geriatric Society (AGS) prioritize facial expressions as a principal pain behavior in older adult populations (7). These guidelines form the primary cornerstone for almost all observational-behavioral pain assessment tools designed for this population group including those living with dementia (8–10). Therefore, all published tools include facial expressions as an essential item or domain in their content (8, 9). For example, facial grimacing (an abstract and non-specific descriptor that is not unique to pain and may also indicate sadness) is part of the Abbey Pain Scale (APS), the Pain Assessment in Advanced Dementia (PAINAD), and the Non-Communicative Patient's Pain Assessment Instrument (NOPAIN) (9). Despite the abundance of such tools that contain facial expressions, only a few have objective facial item descriptors (11). Examples of these are PainChek® and the Pain Assessment Checklist for Seniors with Limited Ability to Communicate-II (PACSLAC-II), both of which contain fine-grained anatomically based facial items (e.g., nose wrinkling), derived from the Facial Action Coding System (FACS) (12–14). The FACS is a catalog of 46 facial action units (AUs), each produced by contraction and/or relaxation of a single or group of facial muscle(s) (13). These discrete facial movements are activated by positive (e.g., joy = AU6+AU12) or negative emotions (e.g., sadness = AU1+AU4+AU15), or pain (e.g., AU4+AU6/7+AU9/10+AU43) in adults (15, 16). It is clear from these AUs that there is an overlap between pain facial behaviors and other emotions. This is complicated by the fact that professional caregivers are not superior in decoding pain facial expressions compared with non-professional caregivers (17). One way of overcoming these difficulties is using technology-enabled pain assessments, such as PainChek® (12).

For observational pain assessment tools, the frequency profile of items has been reported on the basis of evidence obtained from controlled clinical and experimental studies (4). Currently, there is no published real-world data attached to any of the existing pain assessment tools, which makes it difficult to evaluate exactly what items are commonly encountered in those living with cognitive impairment, who are in pain. This lack of real-world assessment data can be addressed where data are collected digitally in clinical practice to be pooled and analyzed on a large scale through the use of a central electronic portal (18). Scoring mechanism systems of observational tools can also affect the profiling of pain behavior items. The majority of the tools have ordinal scoring, which makes the process of item profiling difficult or even insurmountable in some cases. In contrast, checklists and binary ratings of items (e.g., in PACSLAC-II and PainChek®) allow an easier identification of pain behavior patterns including those related to facial expressions (11, 18). PainChek® is the first pain observational assessment tool to utilize the combination of artificial intelligence (AI) and smart automation in a mobile application, allowing pain assessment at the point-of-care (18).

Although facial expressions are widely researched, there is still a significant controversy as to whether there is a prototypical or idiosyncratic expression of pain in PLWD. Further, the frequency of facial pain behaviors in dementia is unknown on a population level. Therefore, this study aims to address these perplexing questions based on real-world clinical data. More specifically, this study aimed to investigate the association of the presence of particular pain-related facial AUs with respect to pain intensity, using an AI-enabled pain assessment system “PainChek®” and to determine whether there is any age- or gender-related impact on these associations in PLWD. In doing so, it sought to address the following four hypotheses:

***Hypothesis 1***: The absence/presence of selected facial AUs is associated with pain scores;
***Hypothesis 2***: Facial AU frequencies increase with pain intensity groups (i.e., no vs. mild vs. moderate vs. severe pain, and low vs. high pain);
***Hypothesis 3***: An association exists between facial AU frequencies and anatomical distribution (upper face vs. lower face)/ and pain intensity groups;
***Hypothesis 4***: Facial expressions associated with increasing pain intensity are not influenced by age or gender.

## Materials and Methods

### Ethical Considerations

The study received ethics approval (HR10/2014) from the Human Research Ethics Committee (HREC) of Curtin University (Bentley, WA, Australia). Permission was also obtained from PainChek Ltd (Sydney, NSW, Australia) to provide the data, which were de-identified to protect confidentiality. This study was deemed low risk in accordance with the National Statement on Ethical Conduct in Human Research (2007), which was updated in 2018, as it used collections of nonidentifiable data and involved negligible risk. The study did not require an informed consent from patients, staff, and providers as the data were de-identified, could not be linked to any personal information, and solely aggregated for the purpose of analysis.

Under the terms of its service agreement, the “Services, the PainChek® Application, the PainChek® Platform and all materials provided to the facility are and remain the intellectual property of PainChek Ltd and all rights not expressly granted to the facility under these Service Terms are expressly reserved to PainChek Ltd”. This allows PainChek Ltd to store, access, modify, disclose, and otherwise use the Aggregated Data for any purpose, under the condition that the facility obtains all necessary consents to facilitate the same.

### Study Design

This research is a population-based observational retrospective study with a data-driven methodology.

### Pain Instrument System: PainChek®

The PainChek® is a multimodal, multi-platform, and hybrid pain assessment tool system, which uses automated recognition and analysis of facial AUs, together with checklists of AGS-based and other recognized pain behaviors to identify and quantify pain in non-verbal adults, especially those living with advanced dementia (12). The system is regulatory cleared by Australia's Therapeutic Goods Administration, Health Canada, Singapore Health Sciences Authority, and European Conformity (CE) for this population (18).

**Figure 1:**
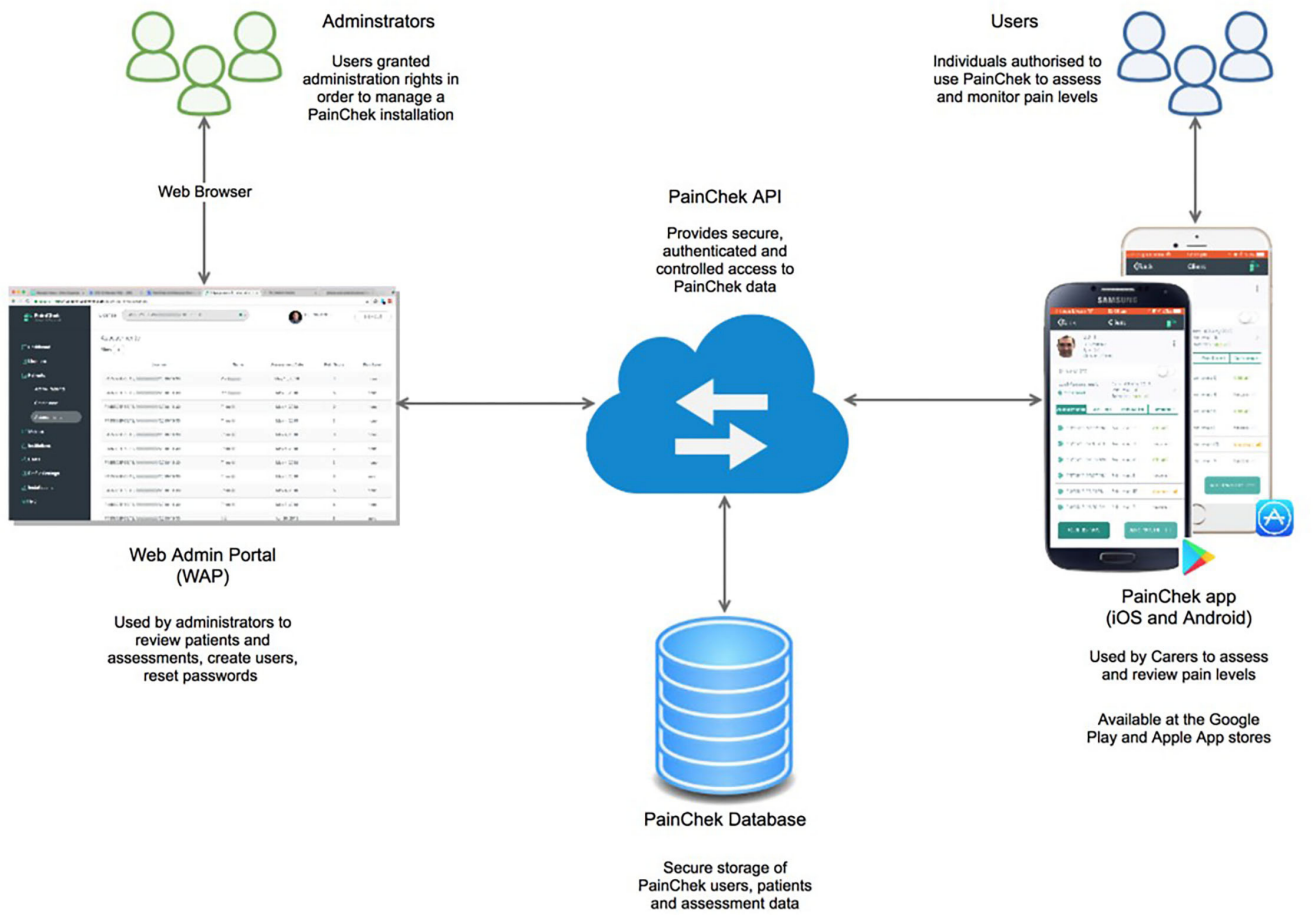
The PainChek® system [internet of things (IoT) connected devices (an App linked to a Web Admin Portal through cloud computing)]. API, application programming interface. Reprinted with permission from PainChek Ltd.

The PainChek® system consists of four components: a point-of-care (POC) smart device enabled application (App) and a web admin portal (WAP), both of which are linked together through cloud computing and internet of things (IoT), PainChek® Application Programming Interface (API), and PainChek® Database (Figure 1) (18).

The PainChek® App comprises of a pain assessment scale, assessments log, pain chart, and comment sections for each patient who was registered and had pain assessments. The PainChek® pain assessment scale is a 42-item instrument, which has items distributed across six domains [Face (9 items), Voice (9 items), Movement (7 items), Behavior (7 items), Activity (4 items), and Body (6 items)]. The *Face* is a FACS-based domain, which has nine pain-relevant AU items representing the most common facial features that are associated with the presence of pain (12, 16, 18, 19). These include upper face AUs: AU4 (brow lowering), AU6 (cheek raising), AU7 (tightening of eyelids), AU9 (wrinkling of nose), and AU43 (closing eyes); and lower face AUs: AU10 (raising of upper lip), AU12 (pulling at corner lip), AU20 (horizontal mouth stretch), and AU25 (parting lips). The *Face* domain uses AI algorithms to detect these facial AUs. The items in each domain are rated on a binary format (present = 1, absent = 0). The pain score is automatically calculated by summing the scores of each domain, and then assigned a pain category based on the following scores: 0–6 (no pain), 7–11 (mild pain), 12–15 (moderate pain), and 16–42 (severe pain) (18). These scores were blindly validated against the APS [a valid and reliable pain assessment tool for people with advanced dementia (20)] by a group of assessors (e.g., health-care professionals) in previous studies (12, 21). The association between pain intensity groups of the APS and PainChek® was evaluated using a contingency table. Cutoff scores were selected as they provided good agreement with the APS with respect to these categories of pain. They were obtained by cross tabulating the raw PainChek® scores against the APS categories, and optimum cutoff scores were obtained in a manner similar to a discriminant analysis (12). The PainChek® pain scale has demonstrated strong psychometric (validity, reliability, internal consistency) and clinimetric (sensitivity, specificity, accuracy) properties in PLWD (12, 21, 22).

The data from the App are synchronized to a web administration portal (WAP). The WAP is an electronic repository platform, which allows central aggregation, storage, and retrieval of pain (assessment) data from the PainChek® App (18). The data collected for each pain assessment include patient demographics, patient unique identifier, facility, facility unique identifier, user demographics, user unique identifier, date and time of assessment, total pain score, pain category, domain scores, and list of items detected in each domain.

### Data Source and Extraction

The de-identified data were extracted by a data custodian (PainChek staff) from the PainChek® database (WAP). The extracted data included demographics of users and patients, date- and time-stamped logs of pain assessments, and the pain scores (total and by domain) and assigned pain intensity categories. The data were extracted for the period from September 10, 2017 (inception date) to March 29, 2019. For this study, we used the *Face* domain of PainChek® to delineate the prevalence and associations of facial expressions with the presence and intensity of pain displayed by PLWD.

### Study Population, Setting, and Context

All users (i.e., aged care home/service staff) received training on the use of the PainChek® tool *via* face-to-face or online training (1.5–2 h) to ensure competency, and to meet the regulatory standards of quality and safety. The users were instructed that PainChek® should not be used on people who are able to provide a reliable self-report of pain, as self-report is the current gold standard; but to be used in people with moderate-to-severe dementia, who are unable to reliably self-report their pain. To ascertain the self-reporting capacity of patients, PainChek® users/assessors were also instructed during training/coaching to ask these patients simple questions, such as “Do you have any pain, aching or soreness?” These questioning strategies were also used in previous validation studies of PainChek® prior to its use by assessors and regardless of patient's cognitive level (12, 21). Staff training was relevant to their setting and consistent with their scope of practice. Following training, each user received at least 10–15 mins of coaching that involved conducting PainChek® assessments on real patients to enhance their confidence with the use of the PainChek® App (18). Face-to-face coaching was either conducted by a clinical specialist from PainChek Ltd, or by a local champion (who had received intensive training) working for the aged care provider.

The sample of PLWD was drawn from two Australian national dementia care programs and 34 Australian residential aged care homes. Pain assessments were conducted during clinical care routines of professional and care staff in residential and community aged care settings while the resident was either at rest (e.g., sitting in a chair or lying in bed) or immediately after movement (e.g., transfer from bed to chair, walking) or participating in routine activities of daily living (e.g., showering).

### Data Analyses

The Statistical Package for the Social Sciences (SPSS, version 26, IBM Corp. 2019) was used for data analyses. Background information including sex (male/female), assessor role, and care home were described using frequency (*f*) and percent (%). The assessor role was broadly categorized as a manager or supervisor, dementia consultant, nurse (enrolled and registered), allied health professional, personal care assistant, and pain nurse. The assessor role was described as a proportion of each category (*f*, %) as well as a proportion of total assessments conducted (*f*, %). Age in years was calculated from the patient's date of birth and their first PainChek® assessment date, and described using mean (M), standard deviation (SD), median (Md), interquartile range (IQR), and minimum and maximum.

The pain scores and *Face* domain scores were right skewed (Kolmogorov-Smirnov) and described using M, SD, Md, and IQR. Hypothesis 1 “AU code frequency increases with increasing pain scores” was examined using the Mann-Whitney U test.

The pain-relevant facial AUs (*f*, %) were described for the total sample followed by four general pain intensity categories based on total PainChek® score [i.e., no pain (0–6), mild pain (7–11), moderate pain (12–15), and severe pain (16–42)] (12). These categories were then simplified into a dichotomized scheme pain as either “low” (no/mild pain; 0–11) or “high” (moderate/severe pain; 12–42) pain groups. The pain group differences for each AU (Hypothesis 2) were examined using Fisher's exact Chi-square (χ^2^) with a two-tailed exact *p*-value reported.

In order to express the magnitude of effect (23), the effect size was computed using Common Language Effect Size (CLES) for non-parametric data, with Cohen's *d* 95% CIs reported (23, 24). The effect size was interpreted as small (0.2), medium (0.5), and large (0.8) (25).

A linear mixed model (LMM) was used to examine the relationship between pain scores and AUs in patients, repeatedly during the analysis period (Hypothesis 3). The LMMs are flexible models that account for correlated errors associated with repeated, continuous, and correlated observations and account for missing data. The LMM examined pain score as a continuous outcome, with fixed effects (Type III sum of squares) age treated as a covariate (Hypothesis 4), with sex (Hypothesis 4) and AUs treated as factors. Potential confounders, aged care home and assessor role, were also included as fixed effects. Patient identifier was set as a random effect with a variance components covariance matrix. A restricted maximum likelihood method of estimation was selected. Model residuals were inspected with no violations noted. Bonferonni corrected pairwise comparisons of estimated marginal means (EMM) were used to further investigate the possible confounding of aged care home and assessor role. In addition, an interaction effect between age care home and assessor role was examined with model fit assessed by Akaike's Information Criterion (AIC), where a lower value indicates an improved model fit. A further LMM analysis with the above specifications was conducted to examine the outcome adjusted total pain score (sum total of the other non-face domains i.e., excluding *Face* domain scores) with (i) total face score (fixed effect) and (ii) upper face score and lower face score (fixed effects).

A binary logistic generalized estimated equation (GEE) with logit link function was used to examine Hypothesis 3 that a high pain category was likely when certain AUs were present as compared with low pain. Age (Hypothesis 4) was treated as a covariate, with sex (Hypothesis 4) and AUs treated as factors, and patient identifier as the repeated subject. For all statistical tests, due to the large sample size, a restrictive *p* < 0.000001 was adopted to assess statistical significance [to reduce the risk of a false positive (26)], with CIs reported. Assessor role and care home factors were not included as these reduced the sample size due to missing data and the model was unable to converge due to some cells having very small samples (<10). A further GEE analysis with the above specifications was conducted to examine the likelihood of a high pain category compared with low pain, with age, upper face scores and lower face scores treated as covariates, sex treated as a factor, and patient identifier as the repeated subject.

## Results

### Data Curation and Processing

A total of 13 cases were removed from the original data set. Two cases were removed from the dataset based on age: one case had an incorrectly entered year of birth, while the other was a test case aged 18 years. An additional 11 test cases were removed, as the assessments were performed by staff not involved in the direct care of patients.

The PainChek® App does not record data on the type of dementia or level of cognitive impairment of the patient, hence these data were not available for the sample. User details (i.e., occupation) were missing for 20.7% of the assessments.

### Sample Demographics

The sample consisted of 3,144 patients with a diagnosis of dementia unable to self-report their pain, aged between 44 and 106 years, and predominantly of female gender (59.0%). Full demographics are described in Table 1.

**Table 1:** Demographic data of study sample.

**Characteristic**	**Statistics**
Sample size, *n* (%)	3,144 (100)
**Age, years**	
Mean (SD)	83.3 (9.0)
Median (IQR)	84.3 (78.4–89.6)
Minimum–Maximum range	43.6–105.9
**Gender**, ***n*** **(%)**	
Female, *n* (%)	1,856 (59.0)
Male, *n* (%)	1,288 (41.0)
**Aged care homes**, ***n*** **(%)**	34 (100)
Bed capacity, mean (range)	86.2 (22–176)
Ownership	
For profit,^a^ *n* (%)	12 (35.3)
Not-for-profit,^b^ *n* (%)	22 (64.7)
Location (remoteness)	
Major cities, *n* (%)	23 (67.6)
Regional, *n* (%)	9 (26.5)
Rural, *n* (%)	2 (5.9)
Location (state)	
Australian Capital Territory, *n* (%)	1 (2.9)
New South Wales, *n* (%)	7 (20.6)
Queensland, *n* (%)	5 (14.7)
South Australia, *n* (%)	3 (8.8)
Victoria, *n* (%)	7 (20.6)
Western Australia, *n* (%)	11 (32.4)
**National dementia care programs**	2 (100)

*IQR, interquartile range*.

a *For-profit (private) providers, including both family-owned, and public companies*.

b *Not-for-profit, including religious, charitable, and community-based organizations*.

### Assessors (Users) Data

All assessments were completed by 389 trained users for patients either at rest or immediately following movement associated with various activities of daily living or physical activity. The majority of users were nurses (35.5%), dementia consultants (26.2%), and personal care assistants (19.3%). Most assessments were conducted by the following users: nurses (44.0%), personal care assistants (20.1%), and dementia consultants (11.6%). Table 2 provides more details on assessors (users) data.

**Table 2:** Assessor details and pain assessments completed.

**Role of assessor**	**Number of assessors *n* = 389**	**Number of pain assessments completed** ***n* = 22,194**
Manager/supervisor, *n* (%)	20 (5.1)	604 (2.7)
Dementia consultant, *n* (%)	102 (26.2)	2,576 (11.6)
Nurse (EN/RN), *n* (%)	136 (35.0)	9,650 (43.5)
Pain nurse, *n* (%)	2 (0.5)	110 (0.5)
Allied health, *n* (%)	9 (2.3)	195 (0.9)
Personal care assistant, *n* (%)	75 (19.3)	4,467 (20.1)
Missing, *n* (%)	45 (11.6)	4,592 (20.7)

*EN, enrolled nurse; RN, registered nurse*.

### Pain Data

#### Overall Sample

There was a total of 22,194 pain assessments completed on the 3,144 patients. The mean number of assessments conducted per patient was 7.1 (SD = 35.7), with 60.8% assessments conducted for females. There was a large variation in the number of assessments per patient, which likely reflected clinical need, duration of institutional care, and/or routine pain assessment protocols within particular care institutions. The patient pain scores ranged from 0 to 35. Table 3 presents the pain data for the sample.

**Table 3:** Pain data of study sample.

**Characteristic**	**Statistics**
**Pain assessments**, ***n*** **(%)**	22,194 (100)
**Pain assessments per patient** ^**a**^	
Mean (SD)	7.1 (35.7)
Median (IQR)	1.0 (1.0–2.0)
**Gender distribution**, ***n*** **(%)**	
Female	13,490 (60.8)
Male	8,704 (39.2)
**Pain scores**	
Mean (SD)	5.0 (4.0)
Median (IQR)	4.0 (2.0–7.0)
Minimum–Maximum range	0–35

*IQR, interquartile range; SD, standard deviation*.

a *Majority of patients had between 1 and 100 assessments conducted (f = 3,106, % = 98.8), with 15 patients having between 101 and 200 assessments (0.5%), and 23 patients having 201 or more assessments (0.7%)*.

#### The Relationship Between Pain Scores/Categories/Groups and Facial AUs

The pain scores for the presence or absence of facial AUs and specific facial AUs according to pain group/intensity category are described in Table 4. For all AUs, the presence of the AU was associated with a significantly higher median pain score (*p* < 0.000001) with medium to large effect sizes (Table 4). The median pain scores when a particular AU was absent was 4.0 for all AUs, except for AU6 (cheek raising), which had a median score of 3.0. The average pain scores when the AU was present ranged from 10.6 to 15.8, with AU20 (horizontal mouth stretch) and AU9 (wrinkling of nose) the highest. For low/high pain categories, during high pain (*n* = 1,712 episodes), AU6 (cheek raising) was the most frequent facial expression (39.7%) detected, followed by AU7 (tightening of eyelids 39.2%) and AU43 (closing eyes 35.4%). In low pain intensities (*n* = 20,482), AU6 (cheek raising) was the most common (24.3%) followed by AU43 (closing eyes 17.1%) and AU12 (pulling at corner lip 16.0%). Large effects were noted for AU9 (wrinkling of nose), AU10 (raising of upper lip), and AU20 (horizontal mouth stretch). Trends were similar when assessed using the adjusted pain score (Table 4).

**Table 4:** Facial action units (AUs) “present” described for total sample, pain score, and low/high pain.

**Facial AUs**	**Total**	**Pain score** ^**a**^		**Effect size**			**Pain dichotomized** ^**b**^		**Adjusted pain score** ^**a**^	
	***f* (%)**	***M (SD)[Md]***					***f*** **(%)**		***M (SD)[Md]***	
		**Absent**	**Present**	**CLES**	***Cohen's d*** **95%CI**		**Low**	**High**	**Absent**	**Present**
*n*	22,194						20,482	1,712		
AU4 brow lowering	2,963 (13.4)	4.6 (3.7) [4.0]	7.5 (4.9) [6.0]	0.70	0.75	0.71–0.79	2,378 (11.6)	585 (34.2)	3.6 (3.4) [3.0]	4.9 (4.3) [3.0]
AU6 cheek raising	5,666 (25.5)	4.4 (3.8) [3.0]	6.5 (4.1) [5.0]	0.65	0.54	0.51–0.57	4,986 (24.3)	680 (39.7)	3.7 (3.5) [3.0]	4.1 (3.7) [3.0]
AU7 tightening of eyelids	3,418 (15.4)	4.5 (3.6) [4.0]	7.6 (4.9) [6.0]	0.72	0.80	0.77–0.85	2,747 (13.4)	671 (39.2)	3.6 (3.3) [3.0]	5.0 (4.3) [4.0]
AU9 wrinkling of nose	1,432 (6.5)	4.7 (3.8) [4.0]	9.0 (5.2) [8.0]	0.78	1.10	1.05–1.16	1,024 (5.0)	408 (23.8)	3.7 (3.4) [3.0]	5.7 (4.6) [5.0]
AU10 raising of upper lip	713 (3.2)	4.8 (3.9) [4.0]	9.0 (5.6) [7.0]	0.77	1.06	0.98–1.13	513 (2.5)	200 (11.7)	3.7 (3.5) [3.0]	5.6 (4.9) [4.0]
AU12 pulling at corner lip	3,796 (17.1)	4.6 (3.8) [4.0]	6.7 (4.3) [5.0]	0.65	0.54	0.51–0.58	3,287 (16.0)	509 (29.7)	3.7 (3.5) [3.0]	4.2 (3.9) [3.0]
AU20 horizontal mouth stretch	698 (3.1)	4.8 (3.8) [4.0]	9.7 (6.1) [8.0]	0.81	1.26	1.18–1.34	456 (2.2)	242 (14.1)	3.7 (3.4) [3.0]	6.6 (5.2) [5.0]
AU25 parting lips	2,885 (13.0)	4.7 (3.8) [4.0]	6.9 (5.0) [5.0]	0.65	0.55	0.51–0.59	2,404 (11.7)	481 (28.1)	3.7 (3.4) [3.0]	4.5 (4.3) [3.0]
AU43 closing eyes	4,117 (18.6)	4.6 (3.7) [4.0]	6.7 (4.6) [5.0]	0.65	0.54	0.51–0.58	3,511 (17.1)	606 (35.4)	3.7 (3.4) [3.0]	4.4 (4.0) [3.0]

*f, frequency; %, percent; M, mean; Md, median; CLES, Common Language Effect Size; SD, standard deviation*.

a *Pain score differences for each AU present vs. absent examined using Mann-Whitney U Test were statistically significant p < 0.000001*.

b *Pain group differences for each AU present vs. absent examined using Chi-square were statistically significant p < 0.000001*.

Similar frequency patterns of upper and lower face AUs were noted in pain categories (*none, mild moderate, and severe pain* groups) (Table 5). Of note, during moderate and severe pain, upper face AUs (AU4, AU6, AU7, AU43) were present more than 30 and 40% in each category, respectively, and were more frequent than lower face AUs (AU9, AU10, AU12, AU20, AU25), which ranged from 9.0 to 28.2% and 16.9 to 37.1%, respectively.

**Table 5:** Facial AUs “present” described for four pain categories.

	**Pain category** ***f*** **(%)**			
**Facial AUs**	**None**	**Mild**	**Moderate**	**Severe**
*n*	16,617	3,865	1,132	580
AU4 brow lowering	1,584 (9.5)	794 (20.5)	345 (30.5)	240 (41.4)
AU6 cheek raising	3,541 (21.3)	1,445 (37.4)	436 (38.5)	244 (42.1)
AU7 tightening of eyelids	1,786 (10.7)	961 (24.9)	389 (34.4)	282 (48.6)
AU9 wrinkling of nose	566 (3.4)	458 (11.8)	246 (21.7)	162 (27.9)
AU10 raising of upper lip	326 (2.0)	187 (4.8)	102 (9.0)	98 (16.9)
AU12 pulling at corner lip	2,344 (14.1)	943 (24.4)	319 (28.2)	190 (32.8)
AU20 horizontal mouth stretch	288 (1.7)	168 (4.3)	119 (10.5)	123 (21.2)
AU25 parting lips	1,735 (10.4)	669 (17.3)	266 (23.5)	215 (37.1)
AU43 closing eyes	2,549 (15.3)	962 (24.9)	357 (31.5)	249 (42.9)

*f, frequency; %, percent*.

*Pain group differences for each AU present vs. absent examined using Chi-square were statistically significant p < 0.000001*.

Overall, the LMM confirmed the significant association between the *Face* domain score (M = 1.16, SD = 1.26, Md = 1.00, IQR = 0.00–2.00) and the adjusted total pain score (excludes *Face* domain score: M = 3.79, SD = 3.54, Md = 3.0, IQR = 1.00–5.00). As the face domain score increased, so did the sub-total pain score (β = 0.23, SE = 0.02, 95% CI: 0.19–0.26, *p* < 0.001). Similarly, when a LMM examined potential associations between upper face and lower face scores, both were positively associated with pain scores (upper face: β = 0.20, SE = 0.02, 95% CI: 0.16–0.25; lower face: β = 0.27, SE = 0.03, 95% CI: 0.21–0.34).

#### Presence of Facial AUs and Predictability of High Pain Scores

The binary logistic GEE odds of reporting a high pain score in the presence of certain AUs are displayed in Table 6. If AU20 (horizontal mouth stretch) was detected, the patients were more than four times more likely to have a documented high pain score, while both AU7 (tightening of eyelids) and AU9 (wrinkling of nose) had almost three times the likelihood of a high pain score. The patients were twice more likely to have a high pain score when AU4 (brow lowering), AU10 (raising of upper lip), or AU25 (parting lips) were present. The presence of AU6 (cheek raising) did not significantly increase the odds of a high pain score (*p* = 0.107). The binary logistic GEE odds of reporting a high pain score for upper face and lower face scores found both were two times more likely to report high pain if present [upper face: Exp(β) = 2.0, 95% CI: 1.9–2.2, *p* < 0.001; lower face: Exp(β) = 2.1, 95% CI: 1.9–2.3, *p* < 0.001].

**Table 6:** Associations between facial action units, age, gender, and high pain scores.

**Model**	**Exp(β)**	**95% CI**		***p*-value**
		**Lower**	**Upper**	
Intercept	0.11	0.03	0.43	0.002
Gender (male)^a^	0.84	0.60	1.19	0.330
Age	0.98	0.97	1.00	0.053
AU4 brow lowering (present)^b^	2.33	1.98	2.75	^*^<0.001
AU6 cheek raising (present)^b^	1.24	0.96	1.59	0.107
AU7 tightening of eyelids (present)^b^	2.81	2.41	3.29	^*^<0.001
AU9 wrinkling of nose (present)^b^	2.71	2.17	3.40	^*^<0.001
AU10 raising of upper lip (present)^b^	2.09	1.66	2.64	^*^<0.001
AU12 pulling at corner lip (present)^b^	1.64	1.36	1.97	^*^<0.001
AU20 horizontal mouth stretch (present)^b^	4.28	3.39	5.41	^*^<0.001
AU25 parting lips (present)^b^	2.26	1.94	2.63	^*^<0.001
AU43 closing eyes (present)^b^	1.76	1.45	2.13	^*^<0.001

*β, standardized coefficient; CI, confidence interval*.

*Binary logistic generalized estimating equation odds of reporting a high pain score when AUs were present*.

a *Compared to female*,

b *compared to absent*,

* *statistically significant at p < 0.000001*.

### Covariates

#### Gender and Facial AUs

The gender was not significantly associated with increasing the odds of a high pain score when facial AUs were detected (Table 6, GEE *p* = 0.330). When examined using a LMM, the gender was not significantly associated with the pain score (LMM *p* = 0.055). However, females showed slightly higher pain scores compared with males (β = 0.27, SE = 0.14, 95% CI: − 0.01–0.55).

#### Age and Facial AUs

In the presence of facial AUs, the age was not significantly associated with increasing the likelihood of a high pain score (Table 6, GEE *p* = 0.053). When examined using a LMM, the age was also not significantly associated with pain scores using a more stringent alpha (*p* = 0.003). Although the pain scores increased with increasing age (β = 0.02, SE = 0.01, 95% CI: −0.01–0.04), the following example demonstrates that it was of little clinical impact. For example, a patient aged 70 years old had a 0.5 unit higher pain score than a patient aged 50 years old.

#### Assessor Role and Aged Care Home/Service

The LMM confirmed an adjustment for confounding by assessor role and aged care home was necessary with some roles associated with higher pain assessment than others. For assessor role, the “consultant” category had the highest estimated marginal mean score (EMM = 12.5, SE = 0.9, 95% CI: 10.8–14.2) compared with other categories, with “nurse” category scoring the lowest (EMM = 9.9, SE = 0.4, 95% CI: 9.1–10.6) (Table 7). Likewise, the assessment of pain varied across the 34 care homes and two programs from a low EMM value of 7.2 (SE = 1.6, 95% CI: 4.1–10.3) up to a high of 19.5 (SE = 3.9, 95% CI: 11.8–27.2).

**Table 7:** The impact of assessor role on pain scores.

**Assessor role**	**EMM**	**SE**	**95% CI**	
			**Lower**	**Upper**
Manager/supervisor	10.9	0.5	10.0	11.9
Dementia consultant	12.5	0.9	10.8	14.2
Allied health professional	10.5	0.9	8.8	12.2
Nurse (EN/RN)^a^	9.9	0.4	9.1	10.6
Personal care assistant	10.1	0.4	9.3	10.9
Pain nurse^a^	11.6	0.4	10.7	12.5

*EMM, estimated marginal means; SE, standard error; CI, confidence interval; EN, enrolled nurse; RN, registered nurse*.

*Linear mixed model (LMM) estimated marginal means for assessor role*.

*LMM reported a fixed effect for assessor category p < 0.000001*.

*Bonferonni pairwise comparisons reported no significant differences using a more stringent alpha*.

a *A significant difference between nurse and pain nurse was noted (p = 0.021) using a traditional alpha p < 0.05 for the Bonferonni pairwise comparisons*.

The LMM investigation indicated the model with an interaction effect between assessor role and care home/service did not reach significance based on the more stringent alpha (*p* = 0.00001); however, it had a better model fit (AIC), and therefore results from this model are reported. Overall, the tests of fixed effects from the LMM showed that each AU was significantly associated with the pain score (*p* < 0.000001).

## Discussion

To date, this is the first and largest clinical study that provides real-world data from pain assessments conducted as part of clinical practice about facial expressions of pain in PLWD. This study aimed to investigate the association of the presence of particular pain-related facial actions (AUs) with respect to pain intensity, using an AI-enabled pain assessment system “PainChek®.” Our findings provide further insight into the impact of age and gender on these facial AUs in this population.

Confirming Hypothesis 3, the AI-based *Face* domain of PainChek® was able to portray distinctive associations of facial expressions with pain intensity groups in PLWD. On an anatomical level, eye-related AUs (AU6, AU7, AU43) were more common than mouth-related AUs and other facial landmarks during *high pain* (i.e., moderate-severe pain). Further, our findings suggest that orbit tightening (AU6/7) is a prominent facial marker of moderate-severe pain. This is not surprising giving that the sensory dimension of pain is primarily encoded in these responsible facial muscle movements and consistent with previous studies (27–29). These findings also suggest that eye-related features may be more dominant and could be a superordinate group during the experience of moderate-severe pain; thus, these are considered as good facial markers of pain intensity. Kunz et al. recently reported that among participants with shoulder pain undergoing passive movement of the joint, the most common cluster of facial expression was “narrowed eyes” related to contracture of AU6/7. The facial activity was displayed in approximately 40% of participants on repeated exposure to movement of their shoulder (19). The authors also reported another cluster of facial activity, which involved AU6/7, AU4 (brow lowering), and AU43 (eye closure) in approximately 10% of participants (“narrowed eyes with furrowed brow and closed eyes”) (19). This is in line with our findings that suggest that the upper face (i.e., eyes and forehead) appears to be the most expressive region of the face in moderate-severe pain.

However, when compared to *low pain* (i.e., no or mild pain), AU20 (horizontal mouth stretch) was the most predictive of moderate-severe pain reporting a large effect, in fact the highest of all AUs, whereas AU25 (lips parting) was also associated with moderate-severe pain. Kunz et al.'s reporting on clusters of facial features exhibited in participants with shoulder pain suggested that approximately 20% of participants exhibited the co-occurrence of AU6/7 (contracture of muscles around the eyes) and AU25/26/27 (mouth opening), which were classified as “narrow eyes and opened mouth” (19). This is not unexpected, given that mouth opening is a means of uttering vocalization during the experience of pain. This also suggests that communicative pain behaviors (i.e., facial and vocal responses) may work in concert to produce synergistic warning signals for both the onlooker and listener.

A recent systematic review of 37 studies reported that lip movements, including AU20 (horizontal mouth stretch), are commonly observed in clinical pain (30). Further, around 80% of these studies found an increase in brow lowering (AU4) and orbit tightening (AU6/7) during pain (30). This concurs with our findings where the presence of these facial AUs was strongly associated with higher pain scores (Table 4), which is congruent with our previous work (31) and that of others (30, 32, 33), confirming our hypotheses 1, 2, and 3.

The predictability of higher pain intensities differed by each AU, with the highest odds being recorded for horizontal mouth stretch (AU20) (four times), followed by eyelids tightening (AU7) and nose wrinkling (AU9) (three times), then brow lowering (AU4), raising of upper lip (AU10), and parting lips (AU25) (two times). The high predicative ability of horizontal mouth stretch (AU20) in combination with parting lips (AU25) underscores the behavioral and dynamic role that the mouth plays as an essential anatomical and functional part of the pain experience. This predictive utility of facial AUs is well established in the literature (15).

Hypothesis 4 was also confirmed with no significant effects for age and gender detected for the frequencies of facial AUs (Table 5), nor were significant interactions for these variables recorded with pain scores or with increasing the likelihood of producing high pain scores. While this is not always the case (34), these findings are in line with Kunz et al. (35, 36), who in their experimental studies found that both age and gender had no interaction effects on the facial expressions of pain. This is also supported by Fuchs-Lacelle, who indicated that both men and women obtain comparable scores on the PACSLAC (37). Despite the differences in pain modalities [i.e., experimental (e.g., temperature, pressure, or weight-induced pain) vs. clinical (e.g., movement associated with activities of daily living)] and pain evaluation method, age and gender were not shown to have an impact on pain facial expressions.

The direct (self-) assessment of pain is often not valid or reliable in advanced stages of dementia (8); and hence it is not considered the gold standard in this population. Thus, the reliance of clinicians and carers on indirect measures, such as observation-based and/or technology-enabled assessments, becomes a necessity. PainChek® uses a hybrid model, incorporating automated facial recognition and analysis (AFRA) and user based completion of digital checklists of observed nonfacial pain behaviors to detect and quantify pain. Given the large number of pain assessments completed and the broad range of PainChek® users across wide geographical area, our findings suggest that this is a feasible approach to pain assessment in the clinical setting. FACS-based analysis is well validated in pain assessment, and it is the current gold standard for assessing facial behaviors across various age groups including older adults with dementia (8, 10, 29, 38, 39). The automation of this process addressed the potential problem of observer bias among health-care providers leading to potential underestimation of pain as highlighted by Prkachin (40).

Using a “big data” approach (i.e., large volume of digitally acquired information) (41), we have demonstrated that anatomically oriented facial expressions are strong and responsive indicators of pain experience in PLWD in everyday clinical practice.

Compared with the previous studies (11, 30), our findings indicate that facial expressions of pain are likely to be both prototypical and idiosyncratic, with more emphasis on the latter in higher pain intensities. Due to the subjectivity of pain experience, interindividual variations of pain-relevant facial AUs may exist (30, 42). Some researchers, however, support the presence of prototypical pain expression, which consists of a certain set of AU constellations (AU4, AU6/7, AU9/10, and AU43) (43). These differences may be attributed to the methodological variations among studies, which include sampling methods (e.g., sampling type, sample size), tools used (e.g., comprehensiveness, item definitions), pain modalities (clinical vs. experimental), and the underlying age, sex, and disease profile of the population being sampled. Unlike other studies, our study design was based on a large set of real-world data. The data were derived as part of the everyday care of residents of aged care facilities during rest and post movement. These residents would have experienced a broad range of conditions that may have contributed to their pain, which is common to this vulnerable population.

Finally, our findings support that the face provides salient and objective nonverbal behavioral information, recruiting certain facial muscles (i.e., AUs) to establish the presence and severity of pain. More specifically, these AUs serve as predictors of the global experience of pain. Importantly, this mechanism is still accessible, operational, and intact even in the absence of verbal report, as the case with our sample (i.e., non-verbal adults with dementia or cognitive impairment).

### Strengths and Limitations

The strengths of this study include the use of a large nationally representative sample, which offered a broad coverage of age and gender demographics across various clinical settings and geographical distributions, and across a range of users. The data were drawn from a central database (i.e., WAP), which provides a rigorous epidemiological approach that streamlines and facilitates the reporting process.

A standardized approach in collecting and analyzing clinical data is critical in order to better understand the impact of pain on facial expressions in the context of dementia and/or cognitive impairment and describe clinically important pain-related facial features in this vulnerable population. The facial descriptors were studied while considering the contribution of other (non-facial) items in the PainChek® pain scale, such as those related to vocalizations, movements, and behaviors. Such an approach supports the notion of multidimensionality and variability of pain experience in non-verbal populations, including those living with dementia. The numbers of reported pain assessments and end users were substantial. Further, the current study that used *in situ* (i.e., real-world) conditions provides evidence of practicality of the use of the tool in various clinical contexts. Recently, the tool has been supportive in recognizing the prevalence of presence and intensity of pain in people with behaviors and psychological symptoms of dementia (4).

The current pain data are not labeled with the type of activity status and do not specify whether they are related to rest- or movement-based assessments; however, all data were collected while the patients were either “At Rest” or “Post Movement” as recommended during user training. The cognitive status and hence the ability to report the pain status of patients was determined by staff completing pain assessments. It is worth noting that while the verbal reporting capacity of patients generally diminishes with the progression of dementia/cognitive impairment, this is not always the case (44). In certain instances, some communicative persons living with moderate to severe dementia may still be able to inform others of their pain experience (45). However, this was confirmed in our study by instructing PainChek® users/assessors during training/coaching to ascertain self-reporting capacity in this group. The subtypes of dementia were not reported and therefore the relationship with dementia subtypes (e.g., Alzheimer's disease, vascular dementia, and frontotemporal dementia) was not evaluated in this study. While this was not covered in our study, it is worth pointing out that the pain experience is likely to be affected by dementia subtypes as their underlying damaged brain regions may distort pain processing pathways (46). This has been demonstrated in experimental and clinical studies such as Cole et al. (47) and Atee et al. (4), respectively. Further, even in our “big data” sample, the epidemiological rarity of certain dementias with reduced pain experience may still be evident, perhaps obscuring any clinically meaningful effect for the experience of pain in these dementia subtypes. A good example of this is frontotemporal dementia, a much less common dementia (compared with other major dementias) that is characterized by the presence of extensive atrophy in the prefrontal cortex, which results in a general reduction of motivational–affective pain experience (46). We also did not study the confounding effects of ethnicity, medications, and medical comorbidities on facial expressions of pain as such data were not available for the analysis. These confounders may have influenced the results. Future studies should address these important areas of research.

In the context of “big data,” there are a number of limitations regarding statistical analyses. For example, *p* values could easily reach statistical significance in large populations (48). However, to counteract this, we employed more stringent *p* values and reported effect size estimates. Statistically, we were also unable to assess the confounding of assessor role and care home factors in the binary logistic GEE models examining *high* and *low pain* categories due to some cells having very small samples (<10). However, the LMM results, which do control for assessor role and care home confounding and allow for missing data, provide more precise estimates of effects for pain. While further investigation is necessary for confirmation, the GEE models were used to highlight those facial features that were more likely to be associated with higher levels of pain. The assessor roles were collapsed broadly into categories for analysis with dementia professionals grouped into their respective professional roles (i.e., dementia nurse categorized as a nurse). These broad categorizations remove the ability to assess the uniqueness inherent to that professional specialization; however, that was not the focus of this study, nor did the sample size for these specializations permit such an analysis.

### Clinical Implications

The facial expressions are powerful indicators and predictors of the pain experience in PLWD that may provide a quick reference and valuable information to time poor clinicians. Further, education and training of clinical staff regarding the patterns of pain facial expressions can improve their detection by increasing their focus on certain facial regions (e.g., upper face), targeting their attention toward a specific facial landmark, for example, the mouth or a small subset of facial actions, and/or discerning the severity of pain in the context of other pain-related behaviors (e.g., vocalizations, movements). These benefits may offer an instrument that facilitates rapid pain detection and hence guides timely clinical decision-making. Thus, an automated analysis of facial AUs in combination with user clinical observations may increase the clinical usability of pain assessment tools through improving identification of pain and therefore its management.

As pain expressions can be formed as a result of a wide range of facial AUs that are commonly involved in positive (e.g., AU6 and AU12 in smiling) and negative (e.g., AU4 in fear or sadness) emotions (11, 13, 38), these findings can be a useful differentiator of these phenomena.

## Conclusions

Using a technology-enabled pain assessment and data-driven approach, we noted specific facial actions associations related to the presence and intensity of pain in PLWD. These observations were independent of age or gender and add to our understanding of facial expressions during clinical pain in PLWD. Furthermore, the study confirms the usefulness of AI-enabled real-time facial analysis during assessment of pain in PLWD in clinical practice (i.e., real-world).

## Data Availability Statement

Deidentified data that does not violate confidentiality can be made available with the approval of PainChek Ltd, upon reasonable request from the date of publication and can only be used for research purposes, and not for product related work, and cannot be shared with a third party. Further inquiries can be directed to the corresponding author/s.

## Ethics Statement

This study was reviewed and approved by Human Research Ethics Committee Curtin University, Perth, Australia. Written informed consent for participation was not required for this study in accordance with the national legislation and the institutional requirements.

## Author Contributions

MA conducted the literature review and drafted the initial version of the article. MA, KH, and JH made substantial contributions to conception, design, and data acquisition. MA and PC conducted the analysis and interpretation of data. All listed authors contributed to the drafts and revisions of the article and approval of the article as written and take responsibility for the content and completeness.

## Conflict of Interest

MA, KH, and JH are co-inventors of the original PainChek® instrument (branded ePAT at the time), which was acquired and subsequently commercialized by PainChek Ltd. (ASX: PCK). They are shareholders of PainChek Ltd. MA previously held the position of a Senior Research Scientist (October 2018–May 2020) at PainChek Ltd., and currently serving the position of Research and Practice Lead at the Dementia Centre, HammondCare. KH is employed as a consultant by PainChek, while also serving as an associate Professor at the University of Prishtina, Kosovo and university associate at the Curtin Medical School, Curtin University, WA, Australia. JH currently holds the position of Chief Scientific Officer at PainChek Ltd., while serving as an Emeritus Professor at Curtin Medical School. The co-inventors had authored a patent titled “A pain assessment method and system; PCT/AU2015/000501” which was assigned to PainChek Ltd. and who have, to date, received granted patents in the jurisdictions of Australia, China, Japan, and the United States. PC was paid as an independent consultant to complete the data analysis for the project. PC is the Principal Consultant of DATaR Consulting providing independent biostatistical services, while also holding adjunct positions at the University of Notre Dame Australia and Edith Cowan University in Western Australia.

## Publisher's Note

All claims expressed in this article are solely those of the authors and do not necessarily represent those of their affiliated organizations, or those of the publisher, the editors and the reviewers. Any product that may be evaluated in this article, or claim that may be made by its manufacturer, is not guaranteed or endorsed by the publisher.

## References

[1] van Kooten J Binnekade TT van der Wouden JC Stek ML Scherder EJ Husebo BS. review of pain prevalence in Alzheimer's, vascular, frontotemporal and Lewy body dementias. Dement Geriatr Cogn Disord. (2016) 41:220–32. 10.1159/000444791 27160163

[2] Molton IR Terrill AL. Overview of persistent pain in older adults. Am Psychol. (2014) 69:197–207. 10.1037/a0035794 24547805

[3] Goucke CR Ed. Pain in Residential Aged Care Facilities: Management Strategies 2nd edition. Sydney: Australian Pain Society. (2018).

[4] Atee M Morris T Macfarlane S Cunningham C. Pain in dementia: prevalence and association with neuropsychiatric behaviors. J Pain Symptom Manage. (2021) 61:1215–26. 10.1016/j.jpainsymman.2020.10.011 33068708

[5] Lautenbacher S Kunz M. Facial pain expression in dementia: a review of the experimental and clinical evidence. Curr Alzheimer Res. (2017) 14:501–5. 10.2174/1567205013666160603010455 27335044

[6] Williams ACD. Facial expression of pain: an evolutionary account. Behav Brain Sci. (2002) 25:439–55. 10.1017/S0140525X02000080 12879700

[7] AGS Panel on Persistent Pain in Older Persons. The management of persistent pain in older persons. J Am Geriatr Soc. (2002) 50:S205–224. 10.1046/j.1532-5415.50.6s.1.x 12067390

[8] Hadjistavropoulos T Herr K Prkachin KM Craig KD Gibson SJ Lukas A. Pain assessment in elderly adults with dementia. Lancet Neurol. (2014) 13:1216–27. 10.1016/S1474-4422(14)70103-6 25453461

[9] Lichtner V Dowding D Esterhuizen P Closs SJ Long AF Corbett A. Pain assessment for people with dementia: a systematic review of systematic reviews of pain assessment tools. BMC Geriatr. (2014) 14:138. 10.1186/1471-2318-14-138 25519741 PMC4289543

[10] Herr K Zwakhalen S Swafford K. Observation of pain in dementia. Curr Alzheimer Res. (2017) 14:486–500. 10.2174/1567205013666160602234526 27335035

[11] Oosterman JM Zwakhalen S Sampson EL Kunz M. The use of facial expressions for pain assessment purposes in dementia: a narrative review. Neurodegener Dis Manag. (2016) 6:119–31. 10.2217/nmt-2015-0006 27032976

[12] Atee M Hoti K Parsons R Hughes JD. Pain assessment in dementia: evaluation of a point-of-care technological solution. J Alzheimers Dis. (2017) 60:137–50. 10.3233/JAD-170375 28800333 PMC5611807

[13] Ekman P Friesen WV Hager JC. (Eds). Facial Action Coding System. Salt Lake City: Research Nexus, Network Research Information (2002).

[14] Chan S Hadjistavropoulos T Williams J Lints-Martindale A. Evidence-based development and initial validation of the pain assessment checklist for seniors with limited ability to communicate-II (PACSLAC-II). Clin J Pain. (2014) 30:816–24. 10.1097/AJP.0000000000000039 24281294

[15] Ekman P Rosenberg EL. What the Face Reveals: Basic and Applied Studies of Spontaneous Expression Using the Facial Action Coding System (FACS). 2nd edition. New York: Oxford University Press (2005).

[16] Prkachin KM Solomon PE. The structure, reliability and validity of pain expression: Evidence from patients with shoulder pain. Pain. (2008) 139:267–74. 10.1016/j.pain.2008.04.010 18502049

[17] Lautenbacher S Niewelt BG Kunz M. Decoding pain from the facial display of patients with dementia: a comparison of professional and nonprofessional observers. Pain Med. (2013) 14:469–77. 10.1111/pme.12050 23369088

[18] Atee M Hoti K Hughes JD. A technical note on the PainChek^TM^ system: a web portal and mobile medical device for assessing pain in people with dementia. Front Aging Neurosci. (2018) 10:117. 10.3389/fnagi.2018.00117 29946251 PMC6006917

[19] Kunz M Prkachin K Solomon PE Lautenbacher S. Faces of clinical pain: Inter-individual facial activity patterns in shoulder pain patients. Eur J Pain. (2021) 25:529–40. 10.1002/ejp.1691 33135324

[20] Abbey J Piller N De Bellis A Esterman A Parker D Giles L. The Abbey pain scale: a 1-minute numerical indicator for people with end-stage dementia. Int J Palliat Nurs. (2004) 10:6–13. 10.12968/ijpn.2004.10.1.12013 14966439

[21] Atee M Hoti K Hughes JD. Psychometric evaluation of the electronic pain assessment tool: an innovative instrument for individuals with moderate-to-severe dementia. Dement Geriatr Cogn Disord. (2017) 44:256–67. 10.1159/000485377 29393207

[22] Hoti K Atee M Hughes JD. Clinimetric properties of the electronic Pain Assessment Tool (ePAT) for aged-care residents with moderate to severe dementia. J Pain Res. (2018) 11:1037–44. 10.2147/JPR.S158793 29910632 PMC5989701

[23] Lakens D. Calculating and reporting effect sizes to facilitate cumulative science: a practical primer for t-tests and ANOVAs. Front Psychol. (2013) 4:863. 10.3389/fpsyg.2013.00863 24324449 PMC3840331

[24] Lenhard W Lenhard A. Calculation of Effect Sizes (2016). Available online at: https://www.psychometrica.de/effect_size.html (accessed November 2, 2021).

[25] Cohen J. Statistical Power Analysis for the Behavioral Sciences. 2nd edition. Hillsdale: Erlbaum Associates (1988).

[26] Benjamin DJ Berger JO Johannesson M Nosek BA Wagenmakers EJ Berk R. Redefine statistical significance. Nat Hum Behav. (2018) 2:6–10. 10.1038/s41562-017-0189-z 30980045

[27] Kunz M Scharmann S Hemmeter U Schepelmann K Lautenbacher S. The facial expression of pain in patients with dementia. Pain. (2007) 133:221–28. 10.1016/j.pain.2007.09.007 17949906

[28] Lints-Martindale AC Hadjistavropoulos T Lix LM Thorpe L. A comparative investigation of observational pain assessment tools for older adults with dementia. Clin J Pain. (2012) 28:226–37. 10.1097/AJP.0b013e3182290d90 21904200

[29] Sheu E Versloot J Nader R Kerr D Craig KD. Pain in the elderly validity of facial expression components of observational measures. Clin J Pain. (2011) 27:593–601. 10.1097/AJP.0b013e31820f52e1 21415714

[30] Kunz M Meixner D Lautenbacher S. Facial muscle movements encoding pain-a systematic review. Pain. (2019) 160:535–49. 10.1097/j.pain.0000000000001424 30335682

[31] Atee M Hoti K Parsons R Hughes JD. A novel pain assessment tool incorporating automated facial analysis: interrater reliability in advanced dementia. Clin Interv Aging. (2018) 13:1245–58. 10.2147/CIA.S168024 30038491 PMC6052926

[32] Lints-Martindale AC Hadjistavropoulos T Barber B Gibson SJ. A psychophysical investigation of the facial action coding system as an index of pain variability among older adults with and without Alzheimer's disease. Pain Med. (2007) 8:678–89. 10.1111/j.1526-4637.2007.00358.x 18028046

[33] Hsu KT Shuman SK Hamamoto DT Hodges JS Feldt KS. The application of facial expressions to the assessment of orofacial pain in cognitively impaired older adults. J Am Dent Assoc. (2007) 138:963–69. 10.14219/jada.archive.2007.0293 17606495

[34] Fillingim RB King CD Ribeiro-Dasilva MC Rahim-Williams B Riley JL 3rd. Sex, gender, and pain: a review of recent clinical and experimental findings. J Pain. (2009) 10:447–85. 10.1016/j.jpain.2008.12.001 19411059 PMC2677686

[35] Kunz M Mylius V Schepelmann K Lautenbacher S. Impact of age on the facial expression of pain. J Psychosom Res. (2008) 64:311–8. 10.1016/j.jpsychores.2007.09.010 18291247

[36] Kunz M Gruber A Lautenbacher S. Sex differences in facial encoding of pain. J Pain. (2006) 7:915–28. 10.1016/j.jpain.2006.04.012 17157778

[37] Fuchs-Lacelle S. Pain and Dementia: The Effect of Systematic Pain Assessment on Clinical Practices and Caregiver Stress. Regina, Saskatchewan: University of Regina (2007).

[38] Rojo R Prados-Frutos JC Lopez-Valverde A. Pain assessment using the Facial Action Coding System. A systematic review. Med Clin-Barcelona. (2015) 145:350–5. 10.1016/j.medcle.2014.08.002 25433779

[39] Craig KD Prkachin KM Grunau RVE. The facial expression of pain. In: Turk DC Melzack R, editors. Handbook of Pain Assessment, 3rd ed. New York: The Guilford Press (2011). p. 117–33.

[40] Prkachin KM. Facial pain expression. Pain Manage. (2011) 1:367–76. 10.2217/pmt.11.22 24645662

[41] Leem JG. Big data and pain. Korean J Pain. (2016) 29:215–6. 10.3344/kjp.2016.29.4.215 27738499 PMC5061637

[42] Lin L-C. Facial expressions as predictors of pain for patients with dementia. Alzheimer's Dement. (2011) 7:S633–4. 10.1016/j.jalz.2011.05.1811

[43] Prkachin KM. The consistency of facial expressions of pain: a comparison across modalities. Pain. (1992) 51:297–306. 10.1016/0304-3959(92)90213-U 1491857

[44] Herr K Coyne PJ Ely E Gélinas C Manworren RC. Pain assessment in the patient unable to self-report: clinical practice recommendations in support of the ASPMN 2019 position statement. Pain Manag Nurs. (2019) 20:404–17. 10.1016/j.pmn.2019.07.005 31610992

[45] Pautex S Michon A Guedira M Emond H Lous PL Samaras D. Pain in severe dementia: Self-assessment or observational scales? J Am Geriatr Soc. (2006) 54:1040–45. 10.1111/j.1532-5415.2006.00766.x 16866673

[46] Scherder EJ Schwaab DF. Pain in dementia. In: Cervero F Jensen TS (eds) Handbook of Clinical Neurology. Amsterdam: Elsevier (2006). p.817–35. 10.1016/S0072-9752(06)80059-X 18808877

[47] Cole LJ Gavrilescu M Johnston LA Gibson SJ Farrell MJ Egan GF. The impact of Alzheimer's disease on the functional connectivity between brain regions underlying pain perception. Eur J Pain. (2011). 15:568-e1. 10.1016/j.ejpain.2010.10.010 21257326

[48] Sloan JA Halyard M El Naqa I Mayo C. Lessons from large-Scale collection of patient-reported outcomes: implications for big data aggregation and analytics. Int J Radiat Oncol Biol Phys. (2016) 95:922–99. 10.1016/j.ijrobp.2016.04.002 27302508 PMC6544440

